# Quality-of-Life- and Cognitive-Oriented Rehabilitation Program through NeuronUP in Older People with Alzheimer’s Disease: A Randomized Clinical Trial

**DOI:** 10.3390/jcm13195982

**Published:** 2024-10-08

**Authors:** Anthia Cristina Fabara-Rodríguez, Cristina García-Bravo, Sara García-Bravo, Isabel Quirosa-Galán, Mª Pilar Rodríguez-Pérez, Jorge Pérez-Corrales, Gemma Fernández-Gómez, Madeleine Donovan, Elisabet Huertas-Hoyas

**Affiliations:** 1 Geriasa Brunete, Nursing Home, 28690 Brunete, Spain; a.fabara.2018@alumnos.urjc.es; 2 Department of Physical Therapy, Occupational Therapy, Physical Medicine and Rehabilitation, Research Group of Humanities and Qualitative Research in Health Science (Hum&QRinHS), 28922 Alcorcón, Spain; jorge.perez@urjc.es; 3 Physiocare Madrid, Physiotherapy Clinic, 28026 Madrid, Spain; sara.garcia.bravo@urjc.es; 4 Department of Physical Therapy, Occupational Therapy, Physical Medicine and Rehabilitation, Universidad Rey Juan Carlos, 28922 Alcorcón, Spain; pilar.rodriguez@urjc.es (M.P.R.-P.); elisabet.huertas@urjc.es (E.H.-H.); 5 PhD Program in Health Sciences, Universidad Rey Juan Carlos, 28922 Alcorcón, Spain; i.quirosa.2016@alumnos.urjc.es (I.Q.-G.); gemma@centrotangram.com (G.F.-G.); 6 TANGRAM, Center for Comprehensive Care for Children and Adolescents, 28032 Madrid, Spain; 7 Independent Researcher, 13409 Berlin, Germany; madeleine.c.donovan@gmail.com

**Keywords:** Alzheimer’s disease, occupational therapy, rehabilitation, NeuronUP, quality of life, cognitive stimulation

## Abstract

(1) **Background:** Alzheimer’s disease (AD) is a progressive neurodegenerative disorder marked by cognitive decline and functional impairment. The NeuronUP platform is a computer program whose main function is cognitive stimulation through three types of activities that change so that the user does not manage to learn it. This program provides opportunities to work on various domains, including activities of daily living (ADLs), social skills, and cognitive functions. The main objective of this randomized clinical trial was to assess the impact of integrating the NeuronUP platform with conventional occupational therapy to enhance or maintain cognitive, perceptual, and quality of life (QoL) abilities in people with AD compared to a control group. (2) **Methods:** A randomized, single-blind clinical trial was conducted. The sample was randomized using a software program, OxMar, which allowed the separation of the sample into a control group (CG) that received their conventional occupational therapy sessions and an experimental group (EG) that received therapy with NeuronUP, in addition to their conventional occupational therapy sessions. An eighteen-week intervention was conducted. (3) **Results:** The study included 20 participants, and significant differences were observed in most variables analyzed, indicating improvements after the intervention, particularly in measures of QoL and cognitive status. (4) **Conclusions:** Our findings demonstrate that an eighteen-week experimental protocol, incorporating the NeuronUP platform alongside conventional occupational therapy, led to improvements in cognitive status and QoL in older adults with AD. Thus, integrating the NeuronUP platform as a complementary tool to occupational therapy can be a valuable resource for enhancing the QoL of individuals with AD. However, due to the small sample size, further studies are needed to corroborate these findings.

## 1. Introduction

Population aging has become a serious social challenge at the global level. Alzheimer’s disease (AD) is a progressive neurodegenerative disease that affects millions of people worldwide. It is considered the leading cause of dementia worldwide and one of the most expensive and lethal diseases of this century [1]. Dementia is considered one of the most important public health challenges in this decade, as it is estimated that by 2030, there will be 74.7 million people with dementia or AD [2]. AD is characterized by the presence of cognitive and functional impairments with a particular onset and course that includes memory loss, difficulties in problem-solving, or changes in behavior and personality [3]. The early stages of the disease are marked by deficits in the ability to encode and store new memories. In the later phases, progressive changes in cognition and behavior occur [1,3]. In addition, reductions in synaptic strength, synaptic loss, progressive neurodegeneration, and metabolic, vascular, and inflammatory changes, together with comorbid pathologies, constitute fundamental elements in the development of AD [3,4].

As the population ages and life expectancy increases, the prevalence of AD rises. Pharmacological treatment helps slow the progress of AD; however, non-pharmacological treatment is the most commonly used as a preventive and therapeutic measure [5]. Non-pharmacological therapies can play a fundamental role in underlining the importance of addressing not only clinical symptoms, but also the quality of life (QoL) of both patients and their caregivers [6].

QoL is defined as ‘an individual’s perception of their position in life in the context of the culture and value system in which they live and in relation to their goals, expectations, standards, and concerns [7]. The concept of QoL in the context of AD includes physical, psychological, and social aspects that affect the overall well-being of individuals [8].

Several studies have shown that interventions aimed at improving QoL can have a positive impact on the management of AD [9]. Cognitive training is a key component of these interventions. This therapeutic approach focuses on activities designed to maintain and improve cognitive functions through specific exercises and has been associated with benefits in memory, attention, and processing speed [10]. Cognitive training has not only shown promise in improving cognitive abilities but also in reducing the psychological symptoms associated with AD, such as depression or anxiety [11]. Cognitive stimulation has been shown to have a positive effect on cognition in people with mild to moderate dementia [2]. In addition, by enhancing patients’ autonomy and coping skills, their QoL can be significantly improved, and the burden on caregivers can be eased [6]. In this context, the role of the occupational therapist is crucial. One of its main functions in the treatment of people with AD lies in stimulating the functional areas of the brain to preserve the person’s cognitive state and improve QoL, both for patients and caregivers [12].

As society progresses, so do new technologies, especially those applied to rehabilitation, and particularly those in fields such as physiotherapy, occupational therapy, and neurological rehabilitation [13]. New technologies can be useful, since digital programs allow greater dynamism, avoiding a learning factor in the user and lack of adherence or motivation towards rehabilitation programs. Likewise, many applications have a record that allows the evolution of task performance to be tracked [14,15].

The NeuronUP platform is a computer program used through tablets or computers whose main function is cognitive stimulation through three types of exercises: games, cognitive stimulation worksheets, and generators (exercises that change so that the user does not manage to learn it) [16,17,18]. This program is currently employed in a wide range of areas, including acquired brain injury, healthy aging, neurodevelopmental disorders, intellectual disability, and mental health. In addition, it covers various areas of intervention within the domain of occupational therapy, such as activities of daily living (ADLs), social skills, and cognitive functions [16,17,18].

The literature frequently highlights the use of new technologies in the care of the elderly; however, there are no previous studies that combine the use of the NeuronUP platform with conventional occupational therapy in people with AD. Therefore, the main objective of this randomized clinical trial was to assess the effect of combining the NeuronUP platform with conventional occupational therapy to enhance or maintain the cognitive, perceptual, and QoL abilities of people with AD compared to a control group. Additionally, the study aimed to assess treatment satisfaction and adherence levels.

## 2. Materials and Methods

### 2.1. Study Design

A randomized, single-blind clinical trial was conducted, with all participants recruited from the GERIASA Brunete nursing home in the Community of Madrid. The study followed the Consolidated Standards Of Reporting Trials (CONSORT) [19] 2010 checklist to ensure methodological rigor.

This project received approval from the Research Ethics Committee of Universidad Rey Juan Carlos (code: 271120234112023, date of the approval: 22 December 2023) prior to its initiation. The ethical principles outlined in the Declaration of Helsinki, first adopted at the 18th Assembly of the World Medical Associatio n (WMA) in 1964 and revised at the 64th General Assembly in Fortaleza, Brazil (October 2013), were adhered to throughout the study. Furthermore, the research complied with current Spanish legislation, including Law 14/2007 on Biomedical Research and Royal Decree 223/2004.

All participants were fully informed of the study’s objectives and provided written informed consent. Lastly, the trial was registered in ClinicalTrials.gov (NCT06499272).

### 2.2. Participants

All patients were selected from the GERIASA Brunete nursing home, located in the Community of Madrid. Eligibility criteria required that patients be over 60 years old and include individuals of both sexes; present a diagnosis of Alzheimer’s disease confirmed by the diagnostic criteria of the International Group for New Research Criteria for the Diagnosis of AD-2010 and pathophysiological markers (amyloid β42, total tau, and phospho-tau in the cerebrospinal fluid, amyloid tracer uptake in positron emission tomography (PET), medial temporal atrophy, and/or fluorodeoxyglucose in structural magnetic resonance imaging) [20]; present mild or moderate cognitive impairment (measured through the MEC-Lobo test with a score greater than 14 points); have adequate manual dexterity to be able to use a tablet; reside permanently in the nursing home; regularly attend occupational therapy treatment at the GERIASA nursing home; and agree to voluntarily participate in the study by signing the informed consent form.

The exclusion criteria for this study were as follows: severe cognitive impairment (assessed using the MEC-Lobo test with a score below 14 points); presence of any physical condition that could compromise the individual’s safety and manual dexterity; and present comorbid diagnoses such as stroke, brain tumors, or heart disease, among others.

The selection process was carried out in collaboration with the medical team at GERIASA Brunete. Medical records of all patients diagnosed with AD were reviewed. Individuals who fulfilled these criteria were invited to participate in the study and were given detailed information regarding the benefits, risks, procedures, and interventions involved to make an informed decision about their participation. Informed consent was obtained from the patients or their legal representatives/caregivers. Participants who consented were asked to sign an informed consent form and were subsequently evaluated by occupational therapists not directly involved in the study.

The OxMaR program (version 2014) was used for minimization and randomization in clinical trials [20] to generate the randomization sequence. Simple randomization was applied, with participants being randomly assigned to the experimental or control group, each with an equal probability [21]. OxMaR generates random sequences, ensuring that the allocation is unpredictable and free from bias. Each assignment was recorded through the software, ensuring a transparent and balanced process.

Participants were randomly allocated to one of two intervention groups, (1) the experimental group (EG), which received a combination of conventional occupational therapy sessions and the NeuronUP platform, and (2) the control group (CG), which received only conventional occupational therapy sessions.

### 2.3. Intervention

The intervention started immediately after randomization and the initial assessment, continuing for a duration of 18 weeks.

Following assignment to the intervention groups, all participants attended occupational therapy sessions. Those in the CG received standard occupational therapy sessions three times a week, each lasting 60 min, aimed at maintaining their cognitive and physical abilities. The conventional occupational therapy sessions, received by both groups, focused on cognitive stimulation, functional activities, psychomotor skills, sensory stimulation, music therapy, and other similar activities. The duration of treatment was 18 weeks, with a total of 54 sessions. Participants in the EG attended conventional occupational therapy sessions three times per week (with the same foci and activities as the CG), with a duration of 30 min plus 30 min of cognitive stimulation with the NeuronUP platform. None of the EG members had previously used the NeuronUP platform.

**Figure 1:**
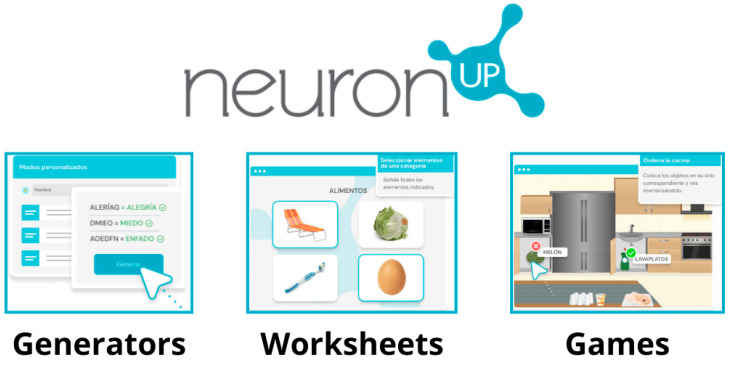
Types of activities on the NeuronUP platform (a screenshot of a tablet).

The NeuronUP sessions for the EG took place in a secure environment. The mode of use of the NeuronUp was through worksheets, generators, and games. The worksheets consisted of cognitive stimulation activities whose difficulty increased based on the user’s results. The generators are activities that constantly change and were used to avoid the feeling of monotony. The games included playful activities designed to enhance cognitive components, such as executive functions [22] (Figure 1).

The protocol developed for the sessions using the NeuronUP platform was as follows:

1. User Reception: Upon entering the occupational therapy room, each user was greeted by the occupational therapist.
2. Tablet Assignment: Each user was given a tablet, where they logged in using their personal profile, previously created in the application.
3. Activity Selection Based on Diagnosis:
  3.1. Initial Activity (10 min):

The session began with an activity where the user had previously demonstrated good performance, aiming to build confidence and motivation.

  3.2. Intervention Activity (20 min):

After completing the initial activity, an activity where the user had faced difficulties (lower assessment scores) was selected. This allowed for targeted work on areas that needed intervention.

Weekly Activity Distribution: Three sessions were held each week, with a different type of activity each day:

- Day 1: Game activities.

Calculation: This activity consisted of addition and subtraction problems that the user had to solve. As the user advanced through the levels, the complexity was increased by advancing the necessary calculation and attention skills.

Correct Name of an Image: In this activity, a picture was displayed, and the user selected the correct name from four options. This targeted language skills and memory.

Odd or Even: Different numbers were shown, and the user determined whether the number was odd or even. This targeted semantic memory.

- Day 2: Worksheets activities.

Repeated Word: In this activity, multiple words appeared on the screen, and the user selected the ones that were repeated. As the levels progressed, more words were introduced, increasing the necessary selective attention and working memory skills.

Select Items from a Category: In this activity, different images were displayed, and at the top, a category (e.g., “food”) appeared. The user selected all the images that belonged to that category. This targeted selective attention and semantic memory.

Arrange Steps of Activities: In this activity, the various steps of an activity were presented in a random order, and the user arranged them in the correct sequence. This task targeted planning, comprehension, and reasoning skills.

- Day 3: Generator activities.

The Butterfly’s Flutter: In this activity, multiple butterflies were displayed on the screen. When the game began, the butterflies started to flap their wings, and the user had to select the last one to move. This exercise targeted selective attention and memory.

The Clone: The screen was divided into two sections, each containing different objects. The task was to quickly find the object that appeared in both sections. This targeted processing speed and selective attention skills.

The Orchestra: In this activity, sound was essential. Before starting, each instrument played individually, allowing the user to associate the sound with the instrument. Then, a melody played, and the user selected the instruments in the order they were heard. This targeted auditory memory and sequencing skills.

Implementation and Supervision: Supervision was conducted by the occupational therapist. During the activities, the therapist monitored users’ performance and progress and provided assistance when needed.

Level-Based Adaptation: Although all users engaged in the same activities, the time spent on each may have varied depending on each person’s individual abilities and pace.

**Figure 2:**
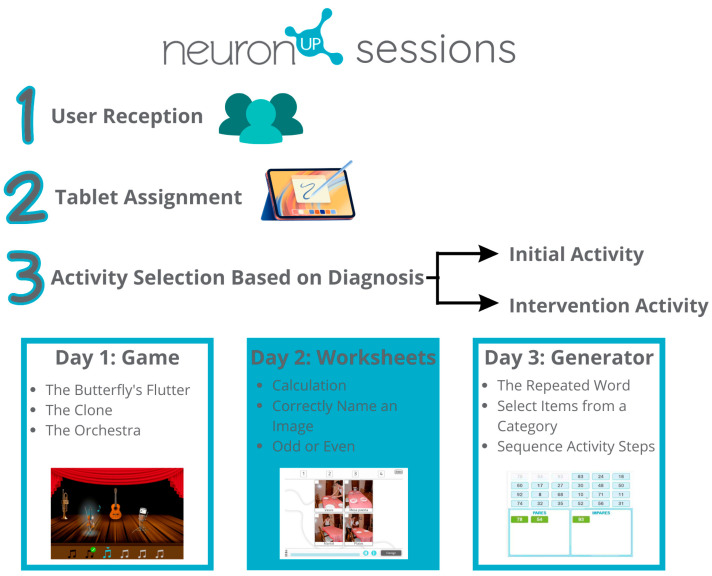
NeuronUP platform session protocol.

The protocol is further detailed below in Figure 2.

### 2.4. Measures

The study started with an initial assessment, during which the following detailed evaluation tests were administered. Upon completing the intervention (at 18 weeks), a second evaluation was conducted to conclude the study. All assessments were applied to both treatment groups in this study.

General Medical Information

Participants’ general health information was collected from the documentation they provided. This included details such as sex, age, medical history, social history, and the number of hours of occupational therapy received per week prior to the study’s onset.

Cognitive Status Assessment

Mini Examen Cognoscitivo-Lobo (MEC): This assessment tool evaluates an individual’s cognitive status. The score obtained may indicate the possible presence of cognitive impairment, with higher scores reflecting better cognitive function [23].

Loewestein Occupational Therapy Cognitive Assessment (LOTCA): A tool adapted and validated for the Spanish population. It assesses cognitive skills in four areas: orientation, visual and spatial perception, visual motor organization, and thought operations. This battery is very useful as it perceives in which cognitive area there are difficulties. Scores range from 1 to 4, except for rational operations ranging from 1–5. The higher the score, the better the cognition [24,25].

Assessment of QoL

WHOQOL-BREF: This self-administered questionnaire evaluates the general perception of QoL and overall health. It consists of four domains: physical health, psychological health, social relationships, and environment. A higher score on this scale indicates a higher QoL [26].

Assessment of Treatment Satisfaction

The Client Satisfaction Questionnaire (CSQ-8): A self-administered survey composed of 8 questions designed to measure satisfaction with the care received, the quality of service, and how well the treatment met the patient’s expectations prior to the intervention. Responses are scored on a scale of 1 to 4, with a maximum total score of 32 points, where higher scores indicate greater satisfaction with the treatment. The Spanish adaptation of the CSQ-8 demonstrates good psychometric properties, preserving the attributes of the original questionnaire, making it a reliable tool for evaluating satisfaction with healthcare services among Spanish-speaking populations. In this study, the CSQ-8 was administered to both treatment groups [27,28].

### 2.5. Statiscal Analysis

Statistical analyses were conducted using SPSS version 28.0 (Copyright© 2013 IBM SPSS Corp., Armonk, NY, USA). The Shapiro–Wilk test confirmed a non-normal distribution of the data. Descriptive statistics were presented as mean and standard deviation for continuous variables, and frequency and percentage for categorical variables. For independent sample analyses, the Mann–Whitney U test was applied. Additionally, the effect size of the differences was estimated by converting Cohen’s d into a correlation coefficient (dr), with values of 0.20, 0.40, and 0.60 indicating small, moderate, and large effect sizes, respectively. For paired sample analyses, the Wilcoxon test was used. Correlations between variables were assessed using Spearman’s rank correlation test. The *p*-value was *p* < 0.05, indicating statistical significance.

## 3. Results

### 3.1. Attrition Rate

**Figure 3:**
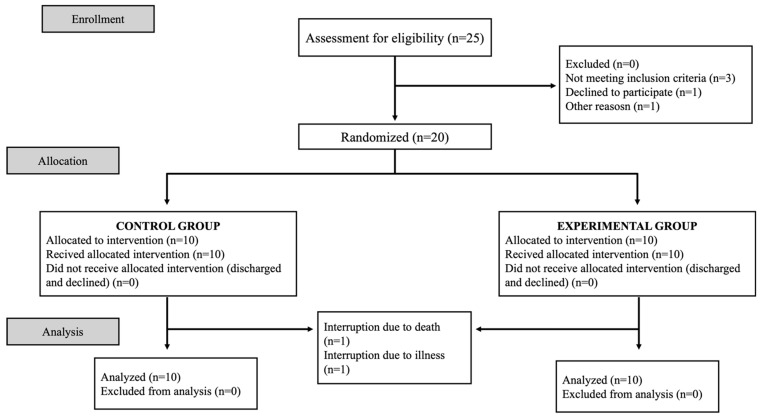
Flow diagram of the experimental procedure.

The study concluded with 20 participants: 10 in the experimental group (EG) and 10 in the control group (CG). Finally, the pre- and post-intervention analysis was carried out with 9/10 individuals from the EG (one dropout in the experimental group due to death) and 9/10 individuals from the CG (one dropout due to serious illness). The flow diagram of the study is shown in Figure 3.

### 3.2. Baseline Characteristics of Participants

The descriptive data of the sample and the administered tests are presented in Table 1. The study included 20 participants, divided into two groups, with a mean age of 81.70 ± 8.35 years. Of the total sample, 70% were female.

According to the data, no significant differences were found at the beginning of the study in the entire sample, indicating that the study started with a fairly homogeneous sample. Differences were found, however, in two variables, QoL Global and Perceptual QoL-G, with the CG being slightly higher than the EG for both variables (Table 1).

When conducting the pre-post intervention analysis between independent samples—CG versus EG—significant differences were found in only two variables analyzed (Table 2), in the MEC-Lobo and in the CSQ-8 variable. This indicates that both groups finished their interventions without apparent changes, except for improvements found at the cognitive level, measured with the MEC-Lobo, and in satisfaction with the intervention, measured with the CSQ-8. Similarly, the effect size reflects values consistent with the significant difference.

An analysis was performed according to the related samples (Table 3), both in the CG and in the EG. The results show that the EG obtained greater changes and significant improvements in more than half of the variables. This suggests that the EG benefited from intervention. The CG only detected a significant difference with respect to the General Perception of QoL, obtaining a lower score after completing the study.

A correlational analysis was conducted to determine how the variables are related with the entire sample (Table 4) in which significant positive relationships are shown between the General Perception of QoL, and the dimensions of global QoL, the physical, psychological, and social scale, as well as the relationship between gender and the dimension of the social scale of QoL.

## 4. Discussion

Currently, there are no published studies in which an intervention with the NeuronUP platform is performed in combination with an occupational therapy treatment in people with AD. The main objective of the present study was to evaluate the effect of the NeuronUP platform in combination with conventional occupational therapy treatment to enhance or maintain cognitive, perceptual, and QoL abilities of people with AD compared to a CG. Our results indicate that the combination of an occupational therapy treatment with the NeuronUP platform, over a period of 18 weeks, can improve the quality of life and cognitive status of people with AD. In this study, high satisfaction and adherence to treatment were obtained in both groups, and these were statistically significantly higher in the experimental group than in the control group. In addition, no adverse side effects were observed during the use of the NeuronUP platform, suggesting that this technology could be employed to improve satisfaction and adherence to traditional interventions in older adults with AD.

In existing studies, there is no clear consensus on the use of the NeuronUP platform for the maintenance or improvement of cognitive abilities. Soldevila-Domenech et al. [29] developed a neurocognitive training protocol through the NeuronUP platform for patients with cognitive impairment and risk of AD occurrence for 12 months. These authors observed improvements in the cognitive functions and functionality of the participants. In addition, a positive correlation of these improvements with QoL, sex, and gender was evidenced. In the study conducted by Mendoza Laiz et al. [18], the effectiveness of cognitive training, as a function of age, was evaluated using a Brain-Computer Interface (BCI) and the NeuronUp platform in people with mild dementia. The results of the study showed that the combined treatment with the NeuronUP platform can prevent, stabilize, or slow cognitive decline in people between 60 and 70 years of age. This suggests that a 5-week protocol with NeuronUP, oriented to daily function in older adults who live independently in their homes and targeting skills including visual perception, spatial orientation, expressive and receptive speech, immediate and logical memory, and image recognition and concepts, can provide greater autonomy and independence for the person. The results of our study are consistent with those of these authors, given that improvements in cognitive functions and QoL were observed in older adults with AD through an 18-week protocol with the NeuronUP platform.

In recent decades, society has become deeply immersed in the use of information and communication technology (ICT), particularly in the fields of health and rehabilitation [30,31]. This shift has brought about a radical transformation in various aspects of daily life. However, the recent and rapid development of these technologies has resulted in a digital divide, with many older adults feeling inept, unable to keep up and adapt to novel platforms [30,31]. Several studies indicate that through the use of ICT, older adults can experience improvements in mental health and social adaptive health [30]. Nevertheless, many authors emphasize that in order to fully benefit from ICT, digital health literacy is essential [31]. To address this potential bias or digital divide, the protocol developed in the present study ensured that participants were always under the supervision of an occupational therapist, and time was allocated beforehand to improve the participants’ digital health literacy.

Cognitive rehabilitation through technology has several benefits compared to traditional cognitive programs. One of the main benefits is that sessions can be designed to be immersive, motivating, and enjoyable, and that it is possible to apply a mix of stimulation, training, and cognitive rehabilitation in the same session [32]. Vincek et al. [33] conducted a review on the impact of new technologies on older adults, concluding that these technologies improve key aspects of QoL, such as autonomy, physical and mental health, social participation, and emotional well-being. The study by Cheng et al. [34] found that cognitive activities through technology-assisted programs improved QoL and emotional well-being in older adults. The results of the study indicated significant improvements in the participants’ overall QoL, demonstrating that technology-assisted occupational therapy can improve QoL in this population. For their part, Zegarra-Ramos et al. [35] added that, with the use of new technologies and gamification, QoL and cognitive function, as well as adherence and motivation toward treatment, can all be improved in older people due to the recreational component. Our results are consistent with those of these previous studies since the participants have reported excellent satisfaction with the treatment through new technologies and gamification.

Improving the cognitive status of people with AD can improve the QoL of patients and caregivers [12]. Caring for a person with AD can place a high burden on families and health systems [36]. A comprehensive approach that enhances QoL can help reduce healthcare costs associated with prolonged care and recurrent hospitalizations resulting from complications linked to advanced cognitive decline and physical dependence. Studies have shown that interventions focused on patient well-being, such as non-pharmacological therapies and psychosocial support programs, can decrease the economic burden on the healthcare system by reducing the need for intensive care or premature institutionalization [37]. Furthermore, improving the QoL for patients with AD directly impacts caregivers, who often experience high levels of stress and emotional overload [37,38]. For example, Lowers et al. [39] developed a qualitative study in which caregivers of people with AD were interviewed. They reported that the QoL of the person with AD, safety, and well-being are the most important issues in their experience, and when these are affected, it can increase the tension of caregivers. Finally, promoting better QoL in patients with AD is a key strategy for fostering healthier aging across the population, which, in the long term, may delay the onset of dependence in older adults [37]. In our study, only the QoL of the participants has been evaluated. It would be interesting, for future studies, to investigate whether caregivers’ QoL is modified with improved QoL and cognitive status of participants with AD through the implementation of the combined occupational therapy treatment and the NeuronUP platform.

Mild cognitive impairment is a precursor to dementia and AD in people over 65 years of age. Therefore, it is important to include healthy aging programs in rehabilitation programs to delay the onset and slow the progression of cognitive decline or AD [2,32,35,40]. Zegarra-Ramos et al. [35] highlighted that the use of video games can help older adults enhance their perception of overall health—both physical and psychological—while also boosting motivation to engage in activities and the rehabilitation process. This, in turn, promotes healthy aging and supports the preservation or improvement of cognitive abilities. Our results are in agreement with those of previous studies since, through gamification and the digitalization of occupational therapy programs, motivation, adherence, and satisfaction with treatment have been improved for people with AD.

This study has several limitations. First, a small sample size was used, so future research should consider a larger sample to evaluate the effects of our experimental protocol. In addition, since the study sample did not present severe cognitive impairment due to the difficulty of using the platform, it would be valuable to carry out future studies to observe the possible benefits in this type of patient through the use of NeuronUP. Likewise, the results are not generalizable to the entire elderly population, given that there are other degenerative diseases, different degrees of cognitive impairment, and varied educational levels. Another limitation of the study is the lack of follow-up measurements over time. It would be advisable to conduct follow-up assessments at one month, three months, and six months to evaluate the long-term effects of the intervention and determine if the improvements are sustained. Finally, all participants were institutionalized in a nursing home, so future studies should examine the benefits in patients living in their own homes.

## 5. Conclusions

Our results demonstrate that an eighteen-week experimental protocol, incorporating the NeuronUP platform alongside conventional occupational therapy sessions, led to improvements in both cognitive status and QoL in older adults with AD. Thus, the NeuronUP platform, when used as a complement to occupational therapy, can be a valuable resource for enhancing the QoL of individuals with AD.

## Figures and Tables

**Table 1:** Baseline descriptive data of the sample (*n* = 20) prior to the intervention.

	Full Sample	Control Group (*n* = 10)	Experimental Group (*n* = 10)	Z	Sig. *p*
Gender (Fr (%)) Female Male	14 (70) 6 (30)	8 (80) 2 (20)	6 (60) 4 (40)		
Age (Mean ± DS)	81.7 ± 8.35	83.1 ± 6.5	80.3 ± 10.03	−0.455	0.649
Lotca (Mean ± DS)	63.7 ± 7.68	64.8 ± 7.45	62.6 ± 8.15	−0.986	0.324
MEC (Mean ± DS)	31.1 ± 2.61	30.3 ± 2.71	31.9 ± 2.37	−1.424	0.154
Percep. QoL-G (Mean ± DS)	81.65 ± 8.75	86.3 ± 9.81	77 ± 4.16	2.160	0.031
QoL Global (Mean ± DS)	3.5 ± 0.82	3.9 ± 0.87	3.1 ± 0.56	−2.091	0.036
Physical scale (Mean ± DS)	25.7 ± 3.37	27.1 ± 2.51	24.3 ± 3.65	−1.763	0.078
Psychological scale (Mean ± DS)	16.7 ± 3.32	18 ± 3.88	15.4 ± 2.11	−1.457	0.145
Social scale (Mean ± DS)	11.1 ± 1.99	11.4 ± 2.59	10.8 ± 1.22	−1.041	0.298
Environment scale (Mean ± DS)	23.1 ± 2.46	23.3 ± 3.09	22.9 ± 1.79	−0.193	0.847

Percep. QoL-G = General Perception of Quality of Life.

**Table 2:** Post-intervention independent sample analysis.

	Control Group (*n* = 10)	Experimental Group (*n* = 10)	Z	Sig. *p*	dr
Lotca (Mean ± DS)	67.7 ± 10.8	70.89 ± 6.48	−0.491	0.623	0.35
MEC-Lobo (Mean ± DS)	30.1 ± 2.18	32.44 ± 2.24	−2.115	0.034	1.05
Percep. QoL-G (Mean ± DS)	81.1 ± 7.65	82.44 ± 5.65	−0.410	0.682	0.19
QoL Global (Mean ± DS)	3.5 ± 1.08	4 ± 1.41	−1.147	0.251	0.39
Physical scale (Mean ± DS)	26.4 ± 3.8	26.44 ± 4.27	−0.041	0.967	0.009
Psychological scale (Mean ± DS)	17.5 ± 3.56	17.78 ± 3.45	−0.534	0.593	0.07
Social scale (Mean ± DS)	11.3 ± 2.31	12.44 ± 1.42	−1.493	0.135	0.59
Environment scale (Mean ± DS)	23.1 ± 3.28	23 ± 2.59	−0.547	0.584	0.03
CSQ-8	27.7 ± 5.31	38 ± 4.92	−3.242	0.001	2.01

Z = Test value.

**Table 3:** Analysis of the intervention’s effect on paired samples.

	Control Group					Experimental Group				
	Pre (Mean ± DS)	Post (Mean ± DS)	Z	Sig.	dr	Pre (Mean ± DS)	Post (Mean ± DS)	Z	Sig.	dr
Lotca (Mean ± DS)	64.8 ± 47.45	67.7 ± 10.8	−0.869	0.385	0.08	62.6 ± 8.15	70.89 ± 6.48	2.136	0.033	1.12
MEC (Mean ± DS)	30.3 ± 2.71	30.1 ± 2.18	−0.541	0.589	0.08	31.9 ± 2.37	32.44 ± 2.24	−1.069	0.285	0.23
Percep. QoL-G (Mean ± DS)	86.3 ± 9.81	81.1 ± 7.65	−2.371	0.018	0.59	77 ± 4.16	82.44 ± 5.65	−2.243	0.025	1.09
QoL Global (Mean ± DS)	3.9 ± 0.87	3.5 ± 1.08	−1.414	0.157	0.40	3.1 ± 0.56	4 ± 1.41	−1.651	0.099	0.83
Physical scale (Mean ± DS)	27.1 ± 2.51	26.4 ± 3.8	0.000	1	0.21	24.3 ± 3.65	26.44 ± 4.27	−1.012	0.311	0.53
Psychological scale (Mean ± DS)	18 ± 3.88	17.5 ± 3.56	−0.712	0.476	0.13	15.4 ± 2.11	17.78 ± 3.45	−2.536	0.011	0.83
Social scale (Mean ± DS)	11.4 ± 2.59	11.3 ± 2.31	−0.414	0.679	0.04	10.8 ± 1.22	12.44 ± 1.42	−2.558	0.011	1.23
Environment scale (Mean ± DS)	23.3 ± 3.09	23.1 ± 3.28	−0.378	−0.378	0.06	22.9 ± 1.79	14.3 ± 2.16	0.000	1	4.33

Z = Test value.

**Table 4:** Correlations between variables.

	Age	Gender	Lotca	MEC	Percp. QoL-G	QoL Global	Physical Scale	Psychological Scale	Social Scale	Environment Scale	CSQ-8
Age	1.000	0.285	−0.433	−0.257	0.098	−0.028	−0.040	0.303	0.134	0.029	−0.025
Gender	0.285	1.000	−0.275	0.048	0.341	0.440	0.297	0.259	0.492 *	0.174	−0.179
Lotca	−0.433	−0.275	1.000	−0.071	−0.048	0.017	−0.294	−0.081	−0.108	0.301	−0.246
MEC	−0.257	0.048	−0.071	10.000	−0.337	−0.046	0.041	−0.059	0.009	−0.067	−0.016
Percep. QoL-G	0.098	0.341	−0.048	−0.337	1.000	0.549 *	0.686 **	0.732 **	0.647 **	0.324	−0.208
QoL Global	−0.028	0.440	0.017	−0.046	0.549 *	1.000	0.412	0.407	0.630 **	0.46	−0.176
Physical scale	−0.040	0.297	−0.294	0.041	0.686 **	0.412	1.000	0.506 *	0.316	0.028	−0.256
Psychological scale	0.303	0.259	−0.081	−0.059	0.732 **	0.407	0.506 *	1.000	0.657 **	0.422	−0.281
Social scale	0.134	0.492 *	−0.108	0.009	0.647 **	0.630 **	0.316	0.657 **	1.000	0.433	−0.038
Environment scale	0.029	0.174	0.301	−0.067	0.324	0.046	0.028	0.422	0.433	1.000	−0.117
CSQ-8	−0.025	−0.179	−0.246	−0.016	−0.208	−0.176	−0.256	−0.281	−0.038	−0.117	1.000

* = *p* < 0.05, ** *p* < 0.01.

## Data Availability

The data presented in this study are available on request from the corresponding author.
